# La pyélonéphrite emphysémateuse à évolution favorable après traitement médical: à propos de 3 observations

**DOI:** 10.11604/pamj.2018.30.223.12086

**Published:** 2018-07-23

**Authors:** Abdelouahed Lasri, Amine Saouli, Othmane Yddoussalah, Tarik Karmouni, Khalid Elkhader, Abdellatif Koutani, Ahmed Ibn attya Andaloussi

**Affiliations:** 1 Service d’Urologie B, CHU Ibn Sina, Faculté de Médecine et de Pharmacie, Université Mohamed V Souissi, Rabat, Maroc

**Keywords:** Pyélonéphrite emphysémateuse, diabète, uroscanner, traitement médical, Emphysematous pyelonephritis, diabetes, uroscanner, medical treatment

## Abstract

La pyélonéphrite emphysémateuse est une infection nécrotique du rein caractérisée par la présence de gaz au sein du parenchyme rénal, des cavités excrétrices ou des espaces péri rénaux. Il s'agit d'une forme grave pouvant engager le pronostic vital. La prise en charge reste controversée: entre l'attitude chirurgicale et le traitement purement médical, il existe une place pour le drainage percutané. Nous rapportons 3 cas de PNE traité par des antibiotiques seuls avec une bonne évolution, nous montrons à travers que le traitement médical pourrait suffire.

## Abstract (en)

Emphysematous pyelonephritis is a necrotic infection of the kidney characterized by the presence of gas within the renal parenchyma, the secretory cavities or the perirenal spaces. It is a severe condition that could be life-threatening. Patient’s management remains controversial and is based either on surgery or on medical treatment. Percutaneous drainage can be also used for the treatment of these patients. We report 3 cases with EPN treated with only antibiotics. Patients’ evolution was favorable. These results show that medical treatment could be sufficient.

## Introduction

La pyélonéphrite emphysémateuse est une infection nécrotique du rein qui met en jeu le pronostic vital en cas de retard thérapeutique. Elle est caractérisée par la présence de gaz au sein du parenchyme rénal, des cavités excrétrices ou des espaces péri rénaux [1,2]. Bien que Kelly et Mac Callum aient décrit le premier cas en 1898, le terme de pyélonéphrite emphysémateuse a été introduit par Schultz et Klorfein en 1962 [2]. Elle survient préférentiellement chez les patients diabétiques ou immunodéprimés, avec une prédominance féminine; et une moyenne d'âge de 57 ans [2]. Le traitement conventionnel de la PNE a été historiquement la chirurgie par le drainage et / ou néphrectomie avec les antibiotiques. La gestion idéale, cependant, reste controversée.

## Patient et observation

Nos observations illustrent la place du traitement médical seul en cas de diagnostic précoce et d'état général conservé.

**Figure 1:**
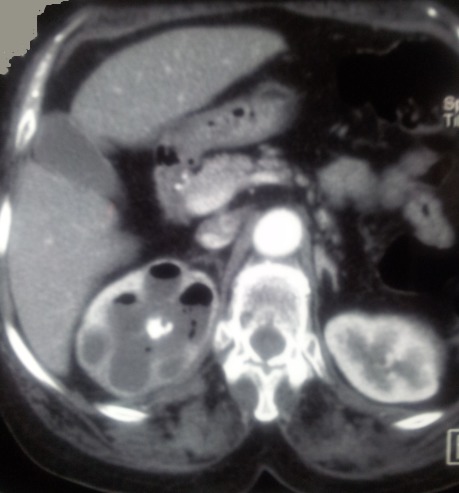
Tomodensitométrie rénale du côté droit (coupe axiale) montrant la présence d'air intra-rénal

**Figure 2:**
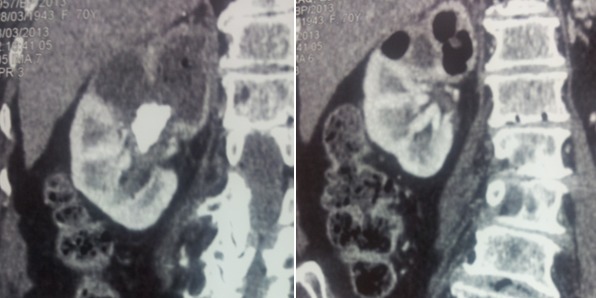
Tomodensitométrie rénale droite (coupe frontale) montrant des images de densité gazeuse avec destruction du pole supérieur en amant d'un calcul pyélique

**Figure 3:**
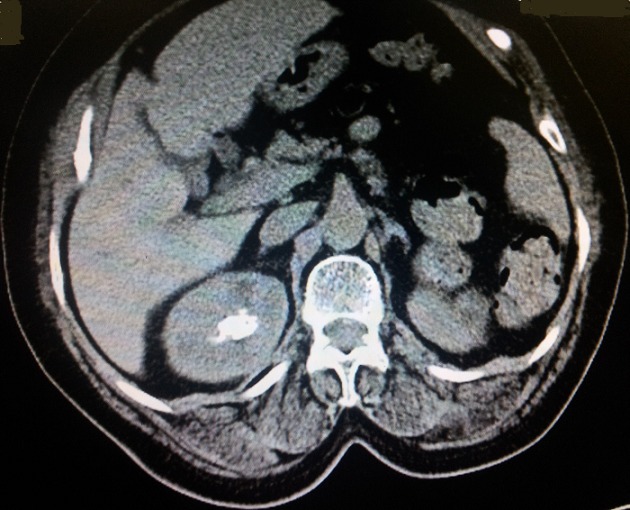
Tomodensitométrie rénale de contrôle (coupe axiale), 2 mois après: disparition des images aériques et liquidiennes

**Observation n°1:** Madame M.L, âgée de 70 ans, connue diabétique sous antidiabétiques oraux depuis 1 an, avec notion d'infections urinaires à répétition depuis 2 ans, a été hospitalisée pour douleur abdominale diffuse, avec des vomissements et une pyurie franche évoluant dans un contexte fébrile depuis une semaine. L'examen clinique a retrouvé une glycémie capillaire à 3g, sans cétonurie, des urines troubles et une sensibilité de la fosse lombaire droite. Sur le plan biologique, une hyper leucocytose était de 22000 GB/mm^3^, une CRP à 95mg/l, l'urée sanguine à 0.85g/l, la créatininémie à 8,9mg/l et la glycémie à 3g/l. L'uroscanner avait mis en évidence la présence de plusieurs images de densité gazeuse polaire supérieure rénale droite en amont d'un calcul obstructif du bassinet faisant 3cm (Figure 1,Figure 2). Le diagnostic de PNE a été retenu, un traitement instauré comportant une insulinothérapie avec une bi antibiothérapie à base de céphalosporines 3^ème^ génération et d'aminoside, une apyrexie a été obtenue après 24 heures, l'analyse d'urine avait montré un Enterobacter Colacae sensible aux quinolones, la patiente a quitté le service après une semaine, sous insuline et quinolones pendant 4 semaines, le scanner de contrôle à deux mois a montré la disparition des images gazeuses (Figure 3).

**Figure 4:**
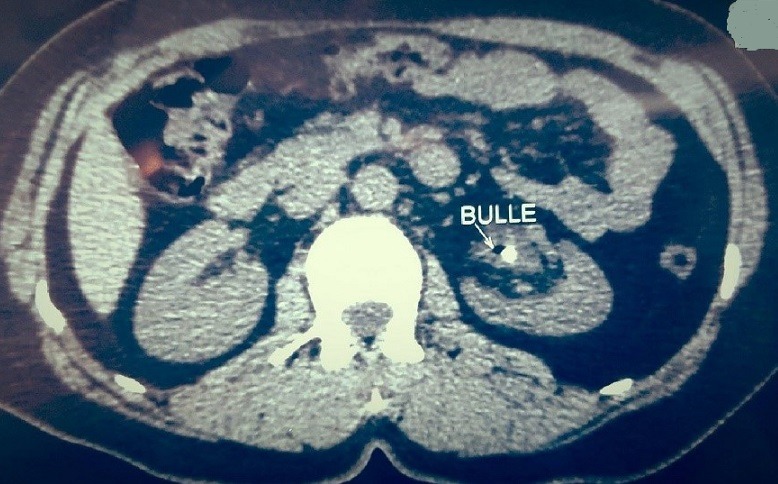
Tomodensitométrie (coupe axiale): une bulle de gaz au niveau du bassinet avec un calcul

**Observation n°2:** Madame E.K, âgée de 55 ans, diabétique depuis 13 ans sous Sulfamides, a été hospitalisée pour des lombalgies gauche fébrile, avec des brûlures mictionnelles, des nausées et des vomissements évoluant depuis 3 jours. L'examen clinique était normal en dehors d'une sensibilité de la fosse lombaire gauche. Le bilan biologique a mis en évidence un syndrome inflammatoire (CRP à 130mg/l, une hyperleucocytose a 18000 GB/mm^3^), une hyperglycémie à 2,5g/l et une fonction rénale correcte. Le scanner abdominale avait montré la présence d'air au sein du bassinet gauche, localisé sans extension au parenchyme avec un calcul de 1,2cm de la jonction pyélourétérale (Figure 4). La patiente a été mise sous insulinothérapie et une association d'antibiothérapie injectable, l'évolution clinique a été favorable, l'apyrexie obtenue après 48 heures et la normalisation des paramètres biologiques au cours de la semaine, l'examen des urines a objectivé une *Escherichia Coli* sensible au Quinolones. La patiente est sortie le 10^ème^ jour, l'antibiothérapie per os a été maintenue pendant 4 semaines. La TDM de contrôle à 6 semaines n'a pas montré d'air, et le calcul a été traité ultérieurement par une LEC.

**Observation n°3:** Madame Z.M, âgée de 52 ans, diabétique depuis 3 ans sous Sulfamides, a été hospitalisée pour des lombalgies droites associées à des brulures mictionnelles et une fièvre. Cliniquement la patiente était en bonne état général, avec un état hémodynamique stable, une fébricule à 38,3 une glycémie capillaire à 2,75g/dl sans cétonurie, la palpation abdominale n'a révélé qu'une sensibilité du flanc droit. La bandelette urinaire était positive, avec au bilan sanguin une hyperleucocytose à 14000 GB/mm^3^, une CRP a 90mg/l, une créatininémie à 10 mg/l et l'urée à 0,67g/l. Le bilan radiologique a comporté une échographie abdominale qui a montré un rein droit discrètement augmenté de volume avec une légère dilatation pyelocalicielle, et un uroscanner qui a mis en évidence des bulles de gaz dans les voies urinaires hautes sans atteinte parenchymateuse ni d'obstacle visible. Une bi antibiothérapie empirique a été rapidement instaurée en parallèle d'une insulinothérapie, l'évolution était favorable avec une apyrexie dès le 2^ème^ jour, la culture des urines a identifié une *Escherichia Coli* sensible aux fluoroquinolones. Le relais par voie orale a duré 4 semaines, le scanner de contrôle à deux mois a montré la disparition des bulles des gaz.

## Discussion

La pyélonéphrite emphysémateuse est une affection rare et grave grevée d'une mortalité oscillant de 7 à 75% selon les séries [3]. Son incidence est en augmentation en raison d'une meilleure connaissance de la maladie, de la diffusion de la tomodensitométrie, ou de l'augmentation de l'incidence du diabète en Asie et dans les pays industrialisés [4]. En effet, il s'agit de patients diabétiques dans plus de 90% des cas, plus volontiers porteurs d'une forme mal équilibrée [5]. Un obstacle à l'écoulement de l'urine, extrinsèque ou intrinsèque est rencontré dans 20 à 40% des cas [6]. Dans la quasi-totalité des cas, les germes responsables sont des bacilles, L'*Escherichia Coli* est retrouvé dans 60% des cas, le Klebsiella Pneumoniae dans 25% des cas. Parfois il s'agit de Pseudomonas ou de Proteus Mirabilis ou Vulgaris [7,8]. La principale hypothèse de la production de gaz est celle de la fermentation intra rénale du glucose. La glycolyse par la voie d'Embden Meyerhof aboutit à la production de pyruvate. L'intervention des entérobacteriaceae entraîne la formation d'acide formique. Ce dernier est converti en dioxyde de carbone et hydrogène par certaines bactéries gazogènes en ambiance acide [1]. La symptomatologie clinique n'est pas spécifique, le tableau habituel est celui d'un syndrome infectieux avec symptomatologie urinaire chez un diabétique [8-10]. La douleur abdominale est souvent au premier plan. L'examen physique met en évidence un empâtement, parfois une rougeur ou une crépitation au niveau du flanc. La pneumaturie est exceptionnelle [11]. L'hyper leucocytose est inconstante. La neuropathie diabétique peut retarder le diagnostic en réduisant la symptomatologie douloureuse et favorise la survenue de formes graves [12]. La radiographie de l'arbre urinaire sans préparation révèle l'emphysème rénal dans 85% des cas ou un rétropneumopéritoine [13]. L'échographie rénale peut montrer des amas de microbulles sous forme de zones hyperéchogènes avec réverbération et atténuation postérieure, mais elle ne permet pas un bilan d'extension précis de la maladie [14]. Le scanner est l'examen le plus performant pour le diagnostic positif et le suivi de la pyélonéphrite emphysémateuse [1] permettant une localisation précise du siège du gaz dans le parenchyme rénal ou le système collecteur, ou dans l'espace péri néphrétique. Huang et Tseng ont établi une classification radiologique à valeur pronostique, conditionnant le choix thérapeutique [15]: 1) stade 1: gaz dans les voies excrétrices seulement. 2) stade 2: gaz dans le parenchyme rénal sans extension dans l'espace extrarénal. 3) stade 3A: extension du gaz ou abcès de l'espace périnéphrétique. 4) stade 3B: extension du gaz ou abcès de l'espace pararénal. 5) stade 4: pyélonéphrite emphysémateuse bilatérale ou sur rein unique.

La pyélonéphrite emphysémateuse est une urgence thérapeutique. Le traitement symptomatique des troubles hémodynamiques, hydro électrolytiques et des dysfonctions d'organes est indispensable et non spécifique, et doit se faire en service de soins intensifs. De la qualité des soins de réanimation dépend en partie le pronostic des patients septiques [1]. Trois principales modalités ont été adoptées pour traiter la PNE: Le traitement médical exclusif. Le traitement médical associé à un drainage percutané ou chirurgical, et le traitement radical qu'est la néphrectomie. Les antibiotiques utilisés doivent être actifs contre les bacilles Gram négatif, administrés à forte dose par voie parentérale et en association synergique. L'antibiothérapie probabiliste initiale associe en général une céphalosporine de troisième génération ou l'imipenème à une fluoroquinolone ou un aminoside. Ce traitement sera adapté aux résultats bactériologiques. Le choix thérapeutique dépend de l'état clinique du patient, de la classification radiologique et de l'existence ou non de facteurs de risques à savoir: la thrombopénie, l'insuffisance rénale aiguë, l'état de choc et les troubles de conscience. Huang (2000), dans sa série de 48 malades (la plus importante de la littérature) avait défini 4 grandes classes radiologiques de la PNE, avec des indications qui ont été adoptées par la majorité des auteurs dans les publications récentes [15]. 1) pour la PNE localisée (classe 1 et 2), le drainage percutané et/ou la levée d'une obstruction combinée au traitement médical donnerait de bons résultats. 2) pour la PNE étendue (classe 3 et 4) avec des manifestations bénignes (moins de deux facteurs de risque), le drainage percutané combiné au traitement médical serait la conduite de 1^ère^ intention. 3) pour la PNE étendue avec une évolution fulminante (deux facteurs de risque ou plus), la néphrectomie fournit les meilleurs résultats et devrait être rapidement réalisée. Des recommandations validées pour un traitement optimal ne sont pas encore établies, mais le traitement conservateur occupe de plus en plus de place du fait du progrès réalisé en matière d'antibiothérapie et des moyens de réanimation, ainsi que dans le domaine de l'imagerie médicale.

## Conclusion

La pyélonéphrite emphysémateuse demeure une infection grave mettant enjeu le pronostic vital et fonctionnel. Cette infection grave doit être suspectée devant tout tableau d'infection urinaire sévère ou ne répondant pas à un traitement médical bien conduit chez un sujet diabétique ou ayant une obstruction des voies urinaires. La TDM est l'examen clé de la PNE, elle a plusieurs intérêts : diagnostique, thérapeutique et pronostique. L'attitude thérapeutique est basée sur les mesures de réanimation, une antibiothérapie adaptée précoce en plus du drainage percutané des collections péri rénales et du drainage des voies urinaires en cas d'obstruction. Le pronostic de la fonction rénale à long terme dépend de degré de destruction parenchymateuse et de l'existence d'une néphropathie associée.

## Conflits d’intérêts

Les auteurs ne déclarent aucun conflit d'intérêts.
